# Non-invasive Resonance Raman Spectroscopy provides an early estimation of depth in a pig model of multi-depth burns

**DOI:** 10.21203/rs.3.rs-6805519/v1

**Published:** 2025-06-08

**Authors:** Rohil Jain, Yanis Berkane, Emmanuella O. Ajenu, Khanh T. Nguyen, Austin Alana Shamlou, Alona Muzikansky, Jonathan Cornacchini, Alexandre G. Lellouch, Basak E. Uygun, Curtis L. Cetrulo, Mark A. Randolph, Korkut Uygun, Padraic Romfh, Shannon N. Tessier

**Affiliations:** Massachusetts General Hospital; Massachusetts General Hospital; Massachusetts General Hospital; Massachusetts General Hospital; Massachusetts General Hospital; Massachusetts General Hospital; Massachusetts General Hospital; Massachusetts General Hospital; Massachusetts General Hospital; Cedars-Sinai Medical Center; Massachusetts General Hospital; Massachusetts General Hospital; Pendar Technologies; Massachusetts General Hospital

**Keywords:** Diagnosis of burn depth, Raman spectroscopy, pig model of burns

## Abstract

Accurate burn depth diagnosis in the early post-burn phase is critical for treatment; however, current assessment methods are subjective, resulting in reduced diagnostic accuracy. Using a compact and portable benchtop setup, we propose a Resonance Raman Spectroscopy protocol to assess burn depth in this phase. By developing a Hemoglobin Index for wounds, we measure hemoglobin in the wound as a marker for wound perfusion, which is altered in burn injuries due to vascular damage. We tested the Hemoglobin Index for wound categorization in a clinically relevant multi-depth burns model in Yucatan pigs. We found a high accuracy of diagnosis on post-burn day 3, an 85% AUC in a binary classifier model for superficial partial-thickness and deep-partial thickness burns, with nearly perfect classification in other categories. Simultaneously, we discuss the potential use of Raman-associated fluorescence in measuring fluorophore deposition in later phases for healing management. Thus, non-invasive measurements with our device may have a high potential for clinical translation.

## Introduction

Burn injuries impact 8.4 million people worldwide, resulting in the loss of more than 7.5 million life years due to disability or death (1). More than 400,000 patients in the United States require medical intervention for severe burns every year, and advances in burn care can significantly enhance the quality of life for these patients (2). Accurate burn depth diagnosis in the acute phase of the injury is vital for optimal treatment of these patients. However, current clinically used assessment methods are subjective and only achieve 60–75% accuracy for even experienced surgeons (3–5). This can delay wound healing and raise the risk of pathological scarring, leading to poor aesthetic and functional outcomes.

Burn wounds are broadly classified into three categories depending on the depth of damage to the skin layers – superficial burns, partial-thickness burns, and full-thickness burns (6). A superficial burn wound experiences damage to only the top epidermal layer of the skin, resulting in erythema (redness), pain, and dryness, with no blistering (7). This type of wound heals within 3–7 days with minimal intervention and no scarring. A partial-thickness (PT) burn wound experiences deeper damage that extends into the dermis and causes blistering, erythema, and severe pain. Healing takes more than 2 weeks and can occur with or without scarring (8). A full-thickness burn wound damages the entire dermis and often extends into the subcutaneous tissue, causing skin discoloration, pain, and a lack of response to pin-prick stimuli (9). Such wounds lose spontaneous healing potential due to the loss of epidermal islets within skin adnexae and extensive damage to the vasculature, causing a loss of blood supply. This necessitates surgical debridement and grafting for healing. In addition to these broad categories, partial-thickness burns are sub-categorized into superficial partial-thickness burns (PT-Superficial) and deep partial-thickness burns (PT-Deep). Although there are similarities in visual appearance and pain levels, PT-Superficial burns are restricted to the papillary dermis, while PT-Deep burns extend into the reticular dermis (10, 11). Consequently, vasculature, collagen, and other structures (such as hair follicles, sebaceous glands) experience different severity of damage. PT-Deep burns may require surgical intervention due to more significant damage that requires longer healing time (12, 13). Further, some wounds can progress to a full-thickness burn due to extensive necrosis in the central ischemic zone, which complicates the surgical procedure (14). This progression highlights the importance of early and accurate assessment of burn depth.

The clinical diagnosis of burn depth currently relies on visual assessment by an experienced surgeon, supplemented by the patient’s subjective report of pain and tenderness (15–17). A histological evaluation of wound biopsies may be performed, but it can be challenging to interpret due to subjectivity in scoring and sampling bias, particularly in partial-thickness burns (17–19). To address these challenges, experimental approaches have explored the quantification of wound perfusion, vascular damage, and denatured collagen across different burn categories as objective markers of burn depth (20). For instance, Laser Doppler Imaging (LDI) captures the differences in perfusion by measuring blood flow in the vasculature, and achieved a 95% accuracy of diagnosis in patients across all burn categories by post-burn Day 3 (21). However, it faces barriers to wide clinical adoption due to extensive device calibration and training requirements, cost limitations, and limited portability (22, 23). Another approach is to use indocyanine green or fluorescein dyes as contrast agents to image vascular damage via diffusion of dyes through the wound (24–26), although there are considerable variations in baseline dye absorption rate between patients and a lack of standardized timing and dosage (27). Spectroscopic techniques such as hyperspectral imaging and near-infrared spectroscopy have also been tested for the quantification of wound water content and hemoglobin for burn depth diagnosis; however, they achieved lower accuracies of diagnosis and remain to be tested in large trials (28–31). Furthermore, none of these approaches can track later phases of the wound healing process, which has implications for scar management (32).

**Figure 1:**
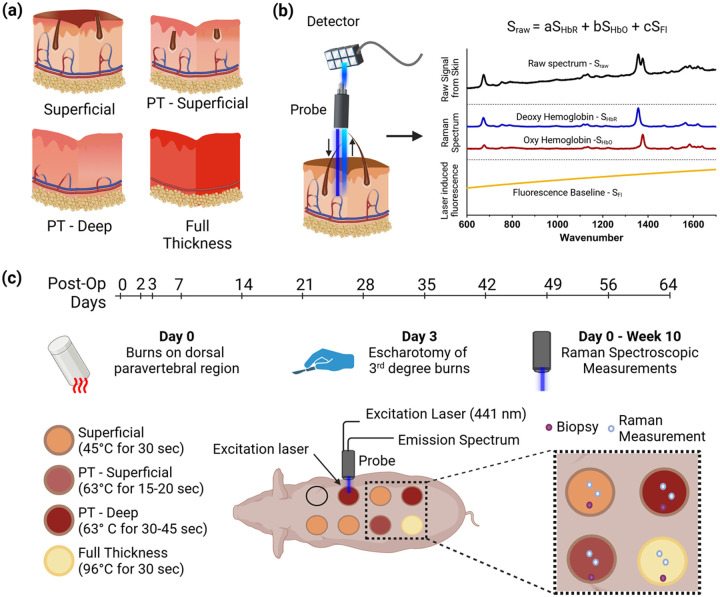
Basis for the diagnostic and plan of experiments. (**a**) Features of damage to vasculature, structures such as hair follicles, and collagen, depending on the depth of damage in each burn category, are shown. Superficial wounds have intact capillaries with low-level damage to the epidermis (shown as a dark top layer in the image). Superficial partial thickness (or PT-Superficial) wounds show loss of epidermis and damage to vasculature in the papillary (upper) dermis. Deep partial thickness (or PT-Deep) wounds show enhanced damage to the vasculature and extensive damage to collagen in both the papillary and reticular (lower) dermis. Full-thickness burns show complete loss of vasculature, hair follicles, and collagen, leading to extensive necrosis. (**b**) A simplified illustration of our Resonance Raman Spectroscopy setup shows a probe and a detector connected via a fiber optic cable. The probe shined the excitation laser (441 nm) and carried the emitted signal to the detector made of a CCD array for processing. The probe was placed in a perpendicular orientation to the wounds for measurements. The emission signal, shown here by the raw spectrum (S_RAW_), consists of two main components – Fluorescence baseline (S_FL_) and the Resonance Raman Signature, which is predominantly the hemoglobin spectrum from oxidized and reduced components (S_HBO_ & S_HBR_, respectively). Individual weights were assigned to each component (denoted by a, b, c) to measure the relative contributions of each component to the raw spectrum. These weights generated the Hemoglobin Index (HI) and Fluorescence Index (FI) for each wound. (**c**) A model of multi-depth burns was created on the back of Yucatan mini pigs to validate the RRS approach. A brass block heated to different temperatures and durations of contact created wounds on Day 0. Each wound was followed on post-burn operation days (POD) 0, 2, 3, 7, and once a week thereafter for 9 weeks (up to POD 64). On POD 3, the surgical escharotomy procedure was performed only on full-thickness wounds. On each assessment day, RRS measurements were performed at two locations on each wound (blue dots). Simultaneously, a biopsy (shown by a purple dot) and a high-quality image were also obtained for each wound.

Here, we propose a Resonance Raman Spectroscopy (RRS) based non-invasive optical measurement approach for accurately categorizing burn depth by post-burn Day 3. This approach targets hemoglobin concentration in wounds by leveraging the resonant amplification of the Raman spectrum of hemoglobin with a 441 nm wavelength laser (33, 34), allowing quantification of even minor differences between wounds with a high resolution. Since burn depth categorization implicitly depends on the blood supply to the wound (Fig. 1a), we hypothesized that RRS-based hemoglobin concentration can aid in the classification of even closely related PT-Superficial and PT-Deep burns on post-burn Day 3. We tested this hypothesis in a clinically relevant Yucatan pig-based model of multi-depth burns (35). We utilized a compact and portable RRS device (**Fig. S1**)(33), to perform non-contact point measurements on the burn wound surface within 3 minutes. The laser was used at a low power (< 10 mW), which is safe for the skin but sufficient to enhance the Raman spectrum from oxidized and reduced hemoglobin (34). The raw emission spectrum from the wounds was analyzed to extract fluorescence information, followed by referencing the remaining Raman spectrum against pre-recorded libraries of hemoglobin for its quantification (Fig. 1b). The resulting hemoglobin concentration from the wound was normalized with healthy skin of the same pig on the same day to develop a *Hemoglobin Index (H.I.)* for wounds. Simultaneously, we also noted interesting preliminary trends in RRS fluorescence, quantified with normalization as *Fluorescence Index (F.I.)* for tracking changes in endogenous fluorophores with a particular interest in the later phases of healing. A total of 24 wounds were created over 3 animals in the superficial, PT-Superficial, PT-Deep, and full-thickness categories. These wounds were assessed weekly for 9 weeks, including more frequent assessments in the first week on post-burn operation days (POD) 2 and 3. We used visual and histological assessments in addition to the RRS measurements (Fig. 1c).

## Results

### Validating a multi-depth model of burns in Yucatan mini pigs to mimic damage in human skin

Burn model creation - Animal models are extensively used for both basic and translation research in burns. Large animal models such as swine are preferred due to similarities with human skin such as thickness, elasticity, the density and distribution of blood vessels, the density of hair follicles, and skin attachment, accurately mimicking features of clinical burn injuries (36). The healing also occurs in 3-phases, including inflammation, proliferation, and remodeling/re-epithelialization (36). However, there can be significant differences in the total healing time between humans and pigs, which may also depend on factors such as age and anatomic location of wounds (37, 38). A recent protocol described the generation of multiple-depth contact burns in Yucatan mini pigs (35), where circular brass blocks were heated to 45, 63, or 96° C temperatures to create different burn depths (**Fig. S2**). In the current study, we adapted this protocol to achieve different depths of partial-thickness burns (superficial and deep), which are the most challenging to diagnose accurately in the clinic, by varying the contact duration with skin at 63° C temperature (shown in Fig. 1c). Out of a total of 14 attempts to create partial thickness burns (superficial vs. deep) using this method, only 3 failed to produce the desired depth and were removed from further analysis (see Methods & **Table S1** for inclusion criteria). To confirm each wound category and sub-categories, we performed a visual assessment of damage till POD 3, along with observing time to healing till the end of the study on POD 64. Simultaneously, we assessed biopsies for histological scoring of burn depth on POD 3 and for observing healing patterns by POD 64 in each category.

**Figure 2:**
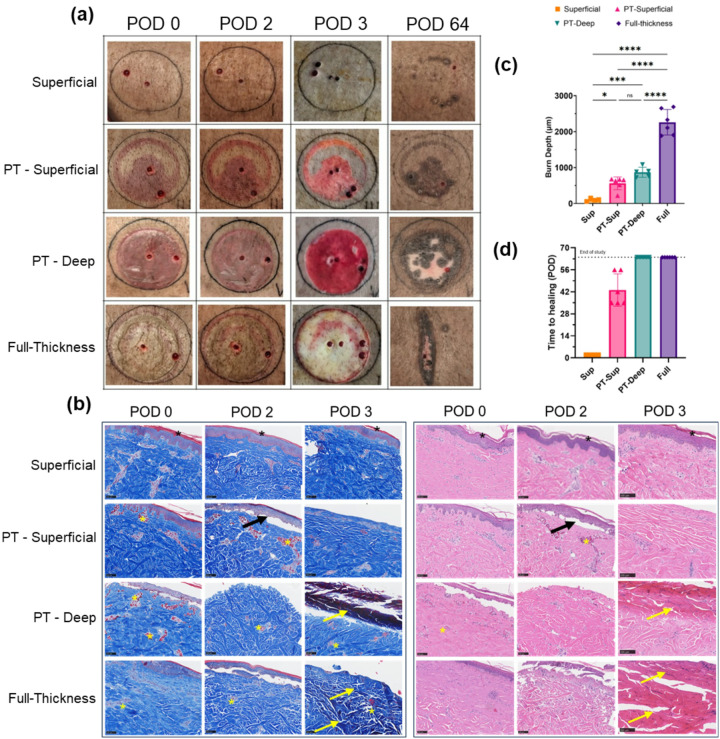
Validation of burn depth with visual and histological assessment. (**a**) High-quality camera images from different days after the burn surgery show features of damage in the early phase (POD 0,2,3) and healing (POD 64). Superficial burns did not show any significant departure from healthy surrounding skin. PT burns in both superficial and deep wound categories showed erythema on POD 0 to 3 and epidermal blistering on POD 0 and 2, which was debrided on POD 3. PT-Superficial wounds healed with re-epithelialization by POD 64, while PT-Deep wounds did not heal completely. Full-thickness burns showed discoloration on POD 0, 2, and 3 before escharotomy. They also showed incomplete healing with contracture by POD 64. (**b**) Microscopic damage features were observed using biopsies with Mason’s Trichrome and H&E stains. Superficial wounds showed intact epidermis (black asterisk). PT-Superficial wounds showed cleaved epidermis (black arrow), with inflammation in the upper dermal layer (yellow asterisk). PT-Deep burns showed severe damage to the upper dermis (dark staining, yellow arrow) and extensive inflammation (yellow asterisk). Full-thickness wounds showed completely damaged dermis with loss of vasculature (yellow arrow, yellow asterisk). Scale bar: 100 μm. (**c**) Burn depth scores from the average of two blinded observers are shown as a bar chart (Mean & SD) with a scatter plot to show values for each wound (n=4 to 6). The significant differences between ANOVA and Tukey’s test-based multiple comparisons are highlighted between burn categories. (**d**) The time to heal for each wound is also shown as a bar chart (Mean) with a scatter plot to show values for each wound (n=4 to 6). Multiple comparisons were not performed due to incomplete healing in the PT-Deep and Full-thickness categories by the end of the study. Asterisks show the following statistical significance levels - **** p < 0.0001, *** p < 0.001, ** p < 0.01, * p < 0.05.

Wound depth categorization with visual assessment - Visual assessment confirmed typical damage features of each burn category in the first few days after injury (POD 0, 2, 3). Superficial burns exhibited mild erythema that resolved by day 2 or 3 without detachment of the epidermis or blisters. On the other hand, partial thickness burns in both the superficial and deep sub-categories exhibited severe erythema that persisted over POD 3. In addition, a thin epidermal blister was formed which was debrided on POD 3 before Raman measurement on the underlying wound bed. The full-thickness burns appeared pale white with a firm, monobloc pinching test, indicating full-thickness necrosis due to vasculature loss, preventing spontaneous healing (Fig. 2a). We thus performed a surgical escharotomy procedure on the necrotic tissue to allow secondary healing. No discernible visual differences between PT-Superficial and PT-Deep wounds were noted until POD 3 (Fig. 2a), highlighting the challenge of visual diagnosis in the acute phase. We followed all wounds for 9 weeks (POD 64). Time to complete healing, defined as complete wound re-epithelization, was recorded. Due to the high clinical relevance of time to healing (13), it has been suggested as a more reliable criterion for sub-categorization into superficial or deep partial-thickness burn categories in animal models (39). Thus, a cut-off time to healing of 8 weeks (POD 56) was set for categorization as a PT-Superficial wound, whereas incomplete healing by 8 weeks was set for categorization as a PT-Deep wound. Based on this cut-off, the observed times to healing were as follows- 2 days for all wounds in the superficial burn category and 43.17 days (range 35 to 56 days) for the PT-Superficial burn category, while complete healing with re-epithelialization was not observed in the PT-Deep and full-thickness burn categories by the end of study on POD 64, as shown in Fig. 2d. We observed significant difference in time to healing among all wound categories, as confirmed by ANOVA with Tukey’s multiple pairwise comparison test (p < 0.05, n = 4–6). However, no significant difference was found between the PT-Deep and full-thickness categories due to incomplete healing in both.

Confirmation of wound depth with histological scoring - Histological evaluation of the different burn categories was performed with Hematoxylin & Eosin (H&E) as well as Mason’s Trichrome (Trichrome) staining of biopsies from POD 0, 2, and 3 (Fig. 2b) due to their ability to differentiate between viable and non-viable tissue as well as different layers of the skin (3, 40). Additionally, burn depth was scored using trichrome-stained biopsies from POD 3 (see Methods). Superficial wounds exhibited minimum damage to the epidermis in the early phase, indicated by a loose stratum corneum layer (Fig. 2b, black asterisk). PT-Superficial burns showed significant damage to the epidermis as observed by the cleavage zone over dermis by POD 2 (Fig. 2b, black arrow), along with vascular damage in the papillary dermis, supported by blood cell staining within dermal capillaries (Fig. 2b, dark red in Trichrome stain & dark pink in H&E, yellow asterisk). This contrasted with the PT-Deep wounds, where vascular damage and collagen denaturation (Fig. 2b, dark blue/purple & dark red in both stains, yellow arrow) were observed both in the papillary as well as reticular dermis with higher severity (darker coloration). The full-thickness wounds displayed very high levels of damage to the vasculature and collagen by POD 3 (Fig. 2b, yellow asterisks & arrows). These histological observations matched the categorization based on the burn creation procedure and visual assessment. Additionally, we quantified burn depth on POD 3 by scoring with two independent blinded scorers as an objective metric for comparison between categories and for correlation with H.I. on POD 3 (**Fig. S3**). Higher histological burn depth scores were observed with higher depth categories as shown in Fig. 2c. The average depth (Mean ± SD) in the superficial burn category was observed to be 102.5 ± 38.95 μm (n = 4). This depth was significantly lower than the PT-Superficial burns, which displayed a depth of 562.9 ±177 μm (n = 6, p = 0.027). The average depth in the PT-Deep category was higher than both previous categories at 870 ± 139.8 μm (n = 5), although it did not show a statistical difference compared to the PT-Superficial category (n = 5, p = 0.15). This may highlight the challenge of using histological burn depth analysis to distinguish between PT-Superficial and Deep sub-categories. Finally, the full-thickness burns had the highest depth scores with an average of 2261 ± 355.1 μm, significantly higher than all the previous categories (n = 6, p < 0.0001). Within the categories that achieved complete healing (i.e. superficial and PT-Superficial burns), we observed a high correlation between time to healing and burn depth (r = 0.91, p = 0.0003; **Fig. S4a**).

#### Hemoglobin Index as a marker for blood perfusion to aid wound categorization by depth

**Figure 3:**
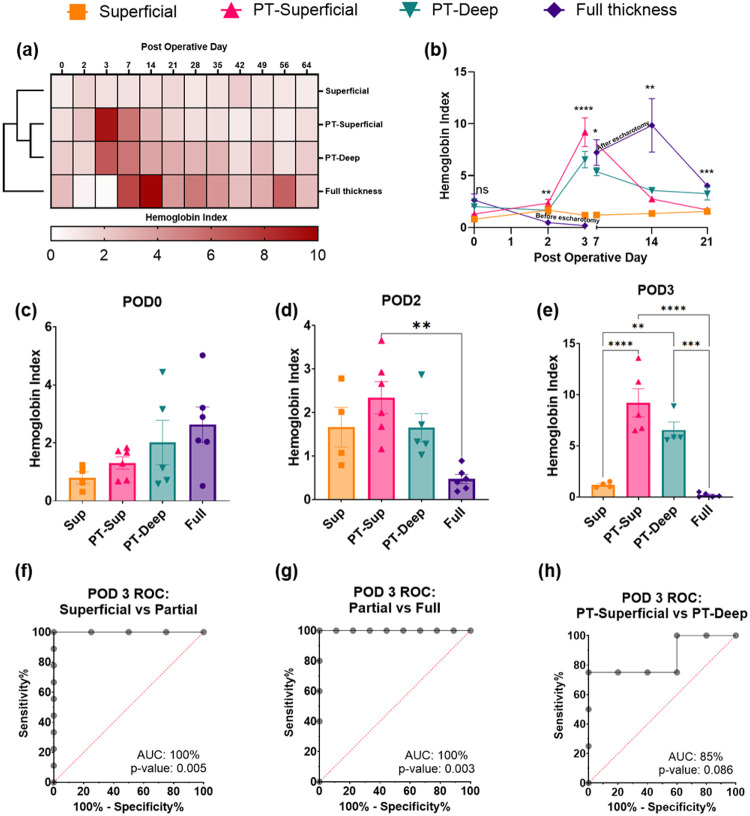
Development of Hemoglobin index (HI) as a biomarker for burn wound perfusion. (**a**) A heatmap provides a broad overview of the variations in HI with wound category (Vertical axis) and assessment time (Horizontal axis). The intensity of the color increases with HI, between the range of 0 and 10. Clustering analysis reveals a close association between PT-superficial wounds and PT-deep wounds, followed by superficial wounds and then full-thickness wounds. (n=4 to 6) (**b**) Variations in HI in the first few weeks (up to POD 21) after the injury are plotted against time by categories. Individual points on the graph show the Mean with Error (SEM) (n=4 to 6). Trends in HI within each wound category can be observed with the connecting lines. Significant results of a One-Way ANOVA for each postoperative day are highlighted with asterisks. (**c**), (**d**), (**e**) HI results are shown as a bar chart (Mean & SD) with a scatter plot to show values for each wound on POD 0, 2, and 3, respectively (n=4 to 6). Significant results of a post-hoc multiple comparison Tukey’s test with ANOVA are highlighted with asterisks. (**f**), (**g**) Show the results of binary classifier models to compare Superficial vs PT (combined superficial & deep) wounds and PT (combined superficial & deep) vs full-thickness wounds on POD 3. HI achieves a near-perfect accuracy of discrimination between these groups (n=4 to 6). (**h**) Shows the results of a binary classifier model to compare PT-Superficial vs PT-Deep wounds on POD 3 (n=4 to 6), with Area Under the Curve (AUC) of a receiver operator characteristic curve and its associated p-value. Asterisks show the following significance levels - **** p<0.0001, *** p<0.001, ** p<0.01, * p<0.05.

Trends in Hemoglobin Index with time and wound category - A portable Resonance Raman Spectroscopy device (33, 41) quantified hemoglobin in the wound by using Hemoglobin Index (HI) on different days of assessment in a non-contact manner. HI was developed as an objective metric to compare hemoglobin concentration across wounds by normalizing the hemoglobin signal intensity from a wound measurement with the hemoglobin intensity from healthy skin measurements from the same animal on the same day (details in the Methods section). This also eliminated the potential measurement biases due to random variations between the RRS devices or systemic differences in hemoglobin levels between different animals on different days. Two measurements close to the center of the wound were obtained and averaged for a single HI per wound per day for all subsequent analyses. Our hypothesis was that HI could be used as an objective and quantitative marker for burn depth categorization by POD 3 because it could accurately measure minor differences in blood perfusion in the wound (by accurate quantification of resonance-enhanced hemoglobin spectrum). Towards this goal, we first plotted the HI results on a heatmap (Fig. 3a), with clustering to identify broad patterns between wound degrees. Each heatmap cell displays the average of all wounds in each category (rows) on a particular day of assessment (columns). Based on the clustering analysis, we observed a close association between PT-Superficial and PT-Deep wounds, followed by superficial burns, and then the full-thickness wounds (Fig. 3a). These associations highlighted the ability of HI to capture wound perfusion similarities between each category over time. We also noted the following trends in Mean ± SEM over time within each category- first, the superficial burns showed no major changes in HI throughout the study (HI ~ 1–2); second, the partial-thickness burns showed high HI in the first week for both PT-Superficial (POD 3 HI = 9.19 ±1.38) and PT-Deep burns (POD 3 HI = 6.54 ± 0.79), before a reduction in the following weeks (POD 21 HI = 1.7 ± 0.25 & 3.26 ± 0.61, respectively); and third, the full-thickness burns showed low HI which further reduced to extremely low level by POD 3 (HI = 0.18 ± 0.21). We used a total of 4 and 6 replicates of each burn category, randomly distributed across 3 pigs for the analysis. This confirmed the loss of wound perfusion due to destroyed vasculature, thus causing them to lose their self-healing potential. This was followed by an escharotomy procedure to remove the dead tissue and allow perfusion and healing from the surrounding healthy tissue, which led to an increase in HI in the following weeks (POD 21 HI = 4.03 ± 0.23). Indeed, the HI remained above healthy baseline levels throughout the study for this category (POD 64 HI = 2.63 ± 0.61). These trends highlight the potential of HI in capturing the underlying pathophysiological processes involved in injury and healing.

To further compare variations over time in the early phase, we plotted HI for each category against time up to POD 21 (Fig. 3b). Each point on this plot shows the average HI (y-axis) for a given wound category (legend) on a given day of assessment (x-axis) with error (SEM). Superficial wounds recorded an average HI of less than 2 on most days (range 0.8 ± 0.2 to 1.67 ± 0.45), indicating only a minor increase from a healthy baseline perfusion. Partial-thickness wounds from the superficial and deep sub-categories showed a rapidly increasing HI from POD 0 to POD 3 (HI = 1.31 ± 0.21 to 9.19 ± 1.38 & 2.02 ± 0.77 to 6.54 ± 0.79, respectively). This may indicate an increase in wound perfusion by POD 3 for these categories compared to superficial wounds. HI decreased in the following weeks for both categories (POD 7, 14, and 21, range 1.7 ± 0.25 to 5.39 ± 0.38), although remaining greater than 1. Finally, the full-thickness burns significantly decreased from POD 0 to 3 (POD 0 HI = 2.63 ± 0.61 to POD 3 HI = 0.18 ± 0.09), which might indicate a significant drop in wound perfusion. As noted, an escharotomy procedure is performed at this stage to remove damaged, non-vascularized tissue and improve secondary healing from the surrounding capillary bed. Indeed, we observed high HI on subsequent days (POD 7 HI = 7.23 ± 1.24, POD 14 HI = 9.85 ± 2.58), indicating a significant increase in perfusion. Results of a one-way ANOVA test to compare HI across burn categories on individual days showed significant differences on each day of assessment (p-values: POD 0 = 0.12, POD 2 = 0.003, POD 3 < 0.0001, POD 7 = 0.02, POD 14 = 0.004, POD 21 = 0.0004, n = 4–6, indicated with asterisks in Fig. 3b).

Hemoglobin Index for diagnosing wound category by POD 3 - Due to our interest in diagnosing wound categories based on HI in the early phase, we further plotted bar charts and performed binary classifier-based comparisons between different categories of wounds on POD 0, 2, and 3 (Fig. 3c–h). For bar charts, we performed a one-way ANOVA with post hoc multiple comparisons using Tukey’s test to understand the statistical significance of the differences between each category on each day. We observed the most statistical difference between wound categories on POD 3 (p < 0.05, n = 4–6, Fig. 3e). Two multiple comparisons were found to be statistically insignificant: PT-Superficial vs. PT-Deep (p = 0.18) and superficial vs full-thickness (p = 0.84). However, the identification of superficial vs full-thickness burns can be done reliably using visual assessment.

The lack of a statistical difference (p = 0.18, n = 4–5) between the PT-Superficial and PT-Deep categories may reflect variability in damage within each category. However, this does not limit its utility as a diagnostic in the clinic where a high binary classification ability may be sufficient in guiding treatment decisions for different thicknesses of partial category burns (such as a go or no-go decision for surgical escharotomy). Thus, we further tested HI with binary classifier models to distinguish between different wound categories. The receiver operating characteristic (ROC) analysis of superficial vs partial-thickness (combined Superficial and Deep) and partial-thickness vs full-thickness burns achieved an area under the curve (AUC) of 1 (Fig. 3f, Fig. 3g; p < 0.01, n = 4–6), suggesting near-perfect discriminative ability with a cut-off HI > 3.54 indicating a partial thickness burn. Most importantly, we observed an AUC of 85% (p < 0.1) for PT-Superficial vs PT-Deep burns, as shown in Fig. 3h. A cut-off HI < 6.58 for PT-Deep burns achieved a 75% sensitivity and 80% specificity of diagnosis.

Finally, we also observed a high correlation of HI with histological burn depth scores (r= −0.86, p < 0.0001) and time to heal (r = 0.86, p < 0.0003). These correlations are visualized with a linear regression analysis on a scatter chart provided in **Fig. S4b and S4c**.

### Tracking wound healing in the later stages of the study with histological analysis and RRS Fluorescence Index

**Figure 4:**
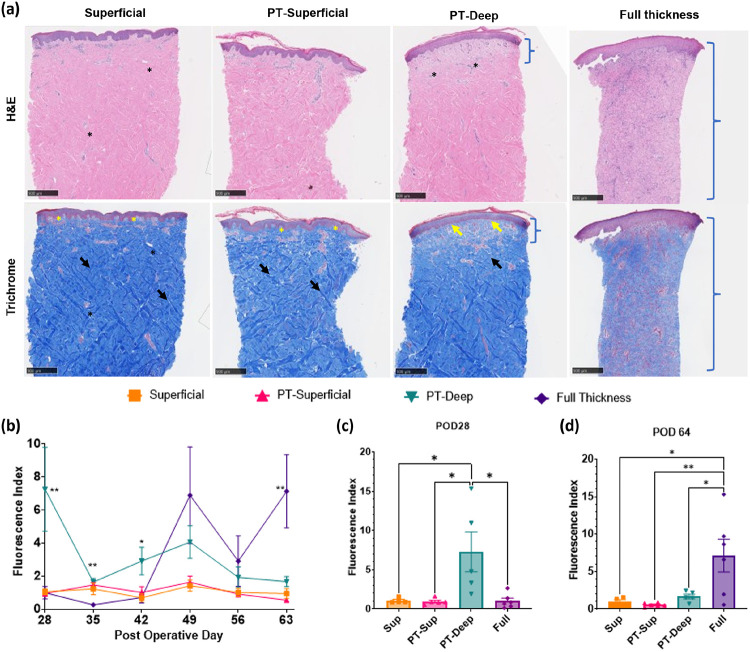
Investigation of wound healing with microscopic analysis of histology and Fluorescence Index (FI). (**a**)Microscopic features of healing are observed using representative biopsies from POD 64 with Mason’s Trichrome and H&E stains. Superficial wounds and PT-Superficial wounds both show intact epidermis with rete ridges (yellow asterisk) and crisscrossed collagen fibers (Trichrome stain, black arrow). PT-Deep burns show mature granulation tissue in the papillary dermis (light staining, blue brackets) with healthy vasculature and erythema (black asterisk), without excessive inflammatory cells and fibroblasts, along with the absence of rete ridges (yellow arrow). Full-thickness burns show granular tissue throughout the biopsy with extensive infiltration of inflammatory cells and fibroblasts (blue brackets). Scale bar: 500 μm. (**b**) FI is plotted against time for each wound category, where individual dots represent the Mean & Error (SEM) with connecting lines to show trends within each category from POD 28 to POD 64 (n=4 to 6). Asterisks highlight the results of a One-Way ANOVA test for each postoperative day. (**c**) FI is shown as a bar chart (Mean & SEM) with a scatter plot to show values for each wound on POD 28 (n=4 to 6). **(d)** FI is shown as a bar chart (Mean & SEM) with a scatter plot to show values for each wound on POD 64 (n=4 to 6). Significant results of a post-hoc multiple comparison Tukey’s test with ANOVA are highlighted with asterisks to show the following significance levels - **** p<0.0001, *** p<0.001, ** p<0.01, * p<0.05.

Histological features of healing- We performed histological staining with H&E and Trichrome (42) of biopsies from the last day of the experiment (POD 64) for all wounds (Fig. 4a). We observed organized patterns of collagen with thick bundling in the superficial wounds (Fig. 4d, bright blue staining in Trichrome, black arrow), with both stains confirming healthy vasculature (Fig. 4a, black asterisk) and presence of epidermal rete ridges (43) (Fig. 4a, finger-like projections into the dermis, yellow asterisk). PT-Superficial burns also showed crisscrossed collagen bundles in the papillary dermis (Fig. 4a, black arrow) with epidermal rete ridges (Fig. 4a, yellow asterisk). However, PT-Deep burns lacked the crisscross pattern (Fig. 4a, black arrow), instead posing a mature granulation tissue (Fig. 4a, blue bracket) with fully formed blood vessels and adnexal structures (Fig. 4a, black asterisk) and the absence of rete ridges (Fig. 4a, yellow arrow).

Trends in RRS Fluorescence Index- Besides the RRS HI in early-phase wound management, RRS fluorescence in the later stages could be useful for quantitatively assessing endogenous fluorophores in the healing wound. Previous studies have shown the potential use of fluorophores such as collagen crosslinks, elastin crosslinks, and NADH as markers for skin conditions, including wound healing (44, 45). RRS fluorescence with the 441 nm laser may reflect the concentrations of these fluorophores due to the excitation peaks of these fluorophores near this wavelength (46). Thus, to explore this further, we developed a Fluorescence Index (FI) to quantify changes in fluorescence in the wounds. FI was derived by normalizing fluorescence values from the wounds with that from healthy skin. This approach allowed us to track FI variations across different wound categories over time, specifically from POD 28 to POD 64 (Fig. 4b).

Our results showed a low mean FI for both superficial and PT-Superficial categories of wounds for all days (range: 0.55 ± 0.1 to 1.63 ± 0.36). In contrast, the mean FI was higher in the PT-Deep category wounds (range: 1.64 ± 0.21 to 7.25 ± 2.54), with a statistically significant difference on POD 28 compared to all other categories of wounds (p < 0.05, n = 4–6, Tukey’s multiple comparisons with ANOVA, Fig. 4c). Additionally, full-thickness wounds showed an increasing FI trend starting on POD 49 (POD42 FI: 0.71 ± 0.33, POD49 FI 6.89 ± 2.92), reaching statistically significant levels compared to all other categories on POD 64 (p < 0.05, n = 4–6, Tukey’s multiple comparisons with ANOVA; Fig. 4d). FI appeared to be higher for full-thickness wounds than PT-deep wounds on POD 64, similar to the granulation tissue thickness (**Figure S5**), warranting a deeper analysis of molecular changes in the granular tissue and their correlation with fluorescent chromophores in a future study.

## Discussion

Our study presents a novel Resonance Raman Spectroscopy (RRS) based non-invasive method to accurately classify burn depth by POD 3, which is an improvement over the existing clinical methods. We measured differences in hemoglobin concentration between wounds by using the resonant enhancement of oxidized and reduced hemoglobin with our RRS device. This enhancement allowed an accurate quantification of the hemoglobin concentration as Hemoglobin Index (HI), which was used to classify the wounds into the superficial, superficial partial-thickness, deep partial-thickness, and full-thickness burn categories. HI-based binary classification for the PT-Superficial vs. PT-Deep categories achieved an Area Under the Curve (AUC) for a Receiver Operating Characteristic (ROC) curve of 85% while achieving a near-perfect classification in the other major burn categories. The RRS device is compact, portable, fast, and safe for the skin. Additionally, it also provided quantitative information regarding fluorophores such as collagen, elastin, and NADH that could be useful to track mid-to-late healing phases. We tested our approach in a Yucatan mini-pig-based novel model of multi-depth burns due to its similarities with human skin.

We established the Hemoglobin Index (HI) as a quantitative marker for blood perfusion in the wound, which is useful for assessing burn depth in the early phase of the injury. In a clinically relevant Yucatan mini-pig model of multi-depth burns, we captured pathophysiological changes in perfusion as an indicator of burn depth (Fig. 1a), observing a high diagnostic ability of HI for categorization of individual burn wounds into different burn categories (Fig. 3c–h). HI achieved near-perfect accuracy in classifying superficial and full-thickness wounds against partial-thickness wounds, with cut-off HI > 3.54 (Fig. 3f & g). It also achieved an AUC of 85% for classifying superficial-partial thickness burns (PT-Superficial) and deep-partial thickness burns (PT-Deep, Fig. 3h) with a cut-off HI > 6.58 for diagnosing PT-Superficial wounds. In contrast, the authors described a 52.5% accuracy in distinguishing burn depth categories using clinical evaluation and 95% accuracy in using Laser Doppler Imaging (LDI) on post-burn day 3 (21). Thus, HI outperformed the current clinical evaluation and provided slightly inferior accuracy to Laser Doppler Imaging (LDI). Our RRS device is smaller and more portable, with fewer setup and calibration requirements than an LDI setup. It also has a smaller probe which allows following body contours, a challenge that can affect LDI measurements (47). In addition, RRS may be useful in multiple phases of wound management with further development of the Fluorescence Index (FI). Overall, RRS measurements are (a) fast, it takes only 3 minutes per measurement with the potential for further reduction in time; (b) performed with a compact and portable setup; (c) quantitative, allowing objective decision-making for surgical debridement and choice of dressings, and (d) possess no safety risks for the patient (48). Thus, this approach is uniquely suited to overcome the barriers in clinical translation observed with LDI, leading to improvements in patient care, including shorter hospital stays, a decrease in wound infection rates, and an overall reduction in the severity of postburn metabolic changes and scarring (49).

Using a standardized burn model creation procedure (Fig. 1c) and stringent criteria for wound inclusion (**Table S1**), we categorized the burns into superficial, PT-Superficial, PT-Deep, and full-thickness burns. This allowed us to capture important pathophysiological differences in healing time (Fig. 2d, ≤ or > 8 weeks), histological features of damage (Fig. 2b, damage to only papillary, vs papillary & reticular dermis), and microscopic features of healing (Fig. 4a, absence vs presence of granulation tissue) between closely related PT-Superficial and PT-Deep burns. Importantly, HI adequately captured these changes in wound perfusion over time between these closely related categories. For instance, by POD 3, the HI was higher for PT-Superficial wounds (HI = 9.185) compared to PT-Deep wounds (HI = 6.538), both of which were higher than the healthy baseline level (HI = 1). This increase from baseline likely represents inflammation-related vasodilation, which helps with wound repair (50). The lower HI for PT-Deep wounds compared to PT-Superficial wounds may indicate reduced perfusion due to greater damage to the vasculature in this category of wounds. This may help in an early prediction and addressing of wound progression of PT-Deep wounds (51). Another instance is the significant drop in HI for full-thickness wounds on POD3 (Fig. 3e, HI- 0.18), which is likely due to extensive vascular damage preventing spontaneous healing (50). Following an escharotomy procedure to improve secondary wound healing and perfusion from the surrounding tissue, we observed high HI on subsequent days (Fig. 3b, POD7 & 14 HI- 7.23 & 9.85, respectively), indicating the escharotomy procedure’s success.

Our model allowed us to track not only the early damage due to burns but may also provide a tool to track healing in the later phases of the study. Tracking healing in the later phases is especially important for PT-Deep and full-thickness burns due to a higher chance of scar formation in these categories (52). Delayed healing, which is characterized by prolonged inflammation and proliferation in the wound bed, can lead to the formation of hypertrophic and keloid scars with excessive collagen deposition in the late re-modeling stage (53). This is confirmed by the presence of granulation tissue at the end of the study (POD 64) in PT-Deep and full-thickness wounds and its absence in superficial and PT-Superficial wounds (Fig. 4a). Thus, the observation of gradually increasing FI (Fig. 4b–d) - by POD 28–42 for PT-Deep wounds and by POD 42–64 for full-thickness wounds - which is likely related to an increase in fluorophores such as collagen, elastin, etc., may be informative regarding the dynamic changes in wound healing for these wounds (54). While longer study durations are required to characterize later phases in the wound healing process, these changes in FI from POD28–64 certainly motivate future studies. In this capacity, future studies could explore the relationship between individual fluorophores in the skin (such as collagen, elastin, NADH) with FI and its implications in the long-term care of burn patients, such as scarring and contractures (32, 52).

The results of our study provide the impetus for a quick clinical translation to address several challenges for patient care and monitoring in both the early phase for diagnostics and the later phases for optimal wound healing. Since HI could predict wound categories even with minor differences in wound perfusion between superficial-partial and deep-partial thickness wounds, RRS HI has a high potential for use as a secondary, more precise, and objective method of evaluation in addition to the primary clinical evaluation, replacing the current approach of waiting for 5–7 days for deep-partial thickness burns (55). This may help in faster decision-making regarding the treatment of such wounds and lead to improved healing outcomes such as contraction and scarring (56). Our non-contact approach may also reduce the dependence on invasive biopsies, which may be delayed, cause additional discomfort, and are often non-conclusive in selecting the best treatment approach. On the other hand, the information regarding wound perfusion at different stages of healing may inform the development of new therapies which focus on early re-vascularization of wounds (57, 58). A combination of RRS HI and FI may also predict transitions in healing phases for all wound types, enabling pharmacological prophylactic treatments to minimize scars (32). RRS FI may also be useful as an objective measurement of scars (59).

Our study has several limitations. As noted, the RRS FI measurements should be spectrally unmixed to quantify individual fluorescent chromophores for a definite biological assessment with these measurements. We did not observe complete healing with scarring of PT-Deep and full-thickness burns by POD 64, limiting the interpretations with respect to the healing outcomes in these wounds and warranting a longer study. There is a significant difference between the burn depth for PT-deep and full-thickness burns. However, patient burns can be more continuous in depth. These limitations will be addressed in future studies.

## Conclusions

We present a novel resonance Raman spectroscopy-based Hemoglobin Index for evaluating burn wound categorization, which is especially useful in the early phase of the injury. It uses a 441 nm wavelength laser with a high absorption coefficient for hemoglobin, producing high-resolution Resonance Raman spectral signatures with high sensitivity for measuring changes in blood perfusion to wounds. Based on these signatures, we could diagnose burn wound categories in a Yucatan mini-pig model of multi-depth burns with high accuracy by post-burn day 3. Specifically, it achieved an 85% AUC in distinguishing between superficial partial-thickness and deep partial-thickness burns, which are the most challenging to categorize in the clinic. It also provides a quantitative assessment of changes in fluorescence chromophores, which may be useful in managing scars. It provides quantitative information within a few minutes and in a non-invasive manner, thus showing the potential to transform the care of partial-thickness burn patients, which represent one-third of all burn-related hospitalizations in the United States (60).

## Materials and Methods

### Animal Use and Care

Animal description and housing - Three Yucatan mini-pigs of approximately 30 kg each were used to create and optimize the multi-depth burns model for Raman assessment in the current study. Yucatan mini-pigs are used frequently for dermatological research due to their skin similarities to human skin, such as comparable epidermal thickness and dermal collagen structure(61, 62). The animals were housed in a controlled environment with a 12-hour light/dark cycle and provided a standard diet and water.

Ethical care and institutional regulations - All the experimental protocols were approved by the Institutional Animal Care and Use Committee (IACUC) of Massachusetts General Hospital (Boston, MA, USA; No. 2021N000271). All experiments were performed in accordance with the ARRIVE (Animal Research: Reporting In Vivo Experiments) checklist. Humane care was provided to the animals, following the National Institute of Health Guide for the Care of and Use of Laboratory Animals.

### Burn Wound Induction, Care, and Eschar Removal

Anesthesia induction and maintenance – Animals were weighed and kept without food but with free access to water for 12 hours before the scheduled burn-making procedure. At the start of the procedure, the animal was sedated via intramuscular injection of Telazol (2–4.4 mg/kg), Xylazine (1–2 mg/kg), and Atropine (0.04 mg/kg). Once sedated, the animal was transported to the operating room. The ear veins were used for the intraoperative administration of fluids and antibiotics. Anesthesia was maintained in the operating room with inhalation of 2–5% isoflurane.

Wound delimitation using tattoos and randomization - The dorsal paravertebral region of the animals was used to create 8 circular burns. The wounds were delimited to a 4.5-diameter region by performing a tattoo-making procedure. Two rows of four wounds each were created with adjacent wounds spaced 7 cm apart (as shown in **Figure S2**). Each wound was numbered sequentially from one to eight, and each number was then assigned to one of the four categories of burns (superficial, superficial partial thickness, deep partial thickness, or full thickness) by a randomization procedure. Randomization helped remove errors due to physiological factors, such as differences in skin thickness, etc., between different locations on the back.

Protocol for inducing burns - The surgical area was prepped using triple scrub with soap, chlorohexidine, and betadine and draped to maintain sterility around the wounds. A cylindrical brass block of weight 1525.5g as described before (35), was used to create the burn wounds. The block was heated to either 45°C, 63°C, or 96°C in a bead bath to create superficial, partial thickness (PT), or full-thickness burns. The block’s temperature was confirmed using a thermometer inserted into a hole at the center of the block. Blocks heated to 63°C temperature were exposed to the pig skin for increasing durations between 15 to 45 seconds to create different depths of damage to the dermis for achieving superficial partial-thickness (PT-Superficial) and deep partial-thickness (PT-Deep) burns (shown in Fig. 1c). The block was placed on the back of the animals and supported by hand for the duration of the procedure, however, with no additional pressure.

Wound dressing and monitoring - Following the induction of burn wounds, the mini pigs were closely monitored for signs of pain, infection, or other complications. Pain management included the administration of transcutaneous fentanyl and *peros* meloxicam, as prescribed by the attending veterinarian. Wound care was performed by regular cleaning and dressing changes to prevent infection and promote healing. The health of the mini pigs was also monitored daily by veterinary staff which included assessments of body weight, skin condition, and overall behavior. Any signs of distress or illness were promptly addressed.

Escharotomy procedure for full-thickness burns – On Day 3, the animal was sedated following a similar procedure as before and transported to the operating room. The wound area was cleaned with soap, chlorohexidine, and betadine and draped sterilely. A surgeon visually identified full-thickness wounds and performed *en bloc* full-thickness escharotomy using a sterile n°15 scalpel blade and Barraya forceps. Next, hemostasis was performed using simple pressure for small vessels and bipolar electric forceps for larger vessels. Following the Raman assessment, each wound area, including the control measurement regions, was dressed with paraffin-impregnated or simple gauze. Finally, Tegaderm Film (3M, Saint-Paul, MN, USA) was used as a secondary dressing.

### Resonance Raman Spectroscopy Setup

Details of the equipment – A portable Resonance Raman Spectroscopy (RRS) system developed for the study. It comprised of a laser of 441 nm wavelength in a compact probe (8 mm × 12 mm × 60 mm), connected with a fiber optic cable to a laser pump. The probe shines the laser on a 2 mm diameter area of the wound. The resulting emitted spectrum from the tissue passed through a bandpass filter to remove the incident wavelength, before being collected at a charge-coupled device (CCD) detector array. The resulting CCD readout was recorded and analyzed using custom LabView software. Furthermore, a 3D-printed adapter was attached at the front of the probe to maintain a constant distance of 9mm between the probe and the surface of the wound and to achieve a localized dark field around the wound. This 3D-printed adapter was further wrapped in black tape, previously confirmed to have no Raman spectrum to avoid interference. Pictures of the Raman device and the adapter are included in **Figure S1**.

Library creation and calibration procedure - We used the following libraries for this analysis: mitochondria reduced and oxidized, and hemoglobin reduced and oxidized due to the possibility of finding these molecules in the pig skin. No additional libraries were required as no additional unknown peaks were observed from the RR spectrum. These libraries were created using procedures described in Perry et al (33). Cosmic noise was recorded once a month by placing the probe in a completely dark chamber for subsequent subtraction from the raw spectrum. Furthermore, the spectrometer was designed to minimize mechanical and thermal drift and correct spectrometer and laser wavelength drifts using an internal acetaminophen standard.

Spectroscopy algorithm – The 441 nm laser uses a power setting of 8.9 mW. Each measurement was at least 180 seconds long. The RR spectra were collected approximately every second, and a rolling 180-second window was used to create the average raw spectrum to minimize random noise. This spectrum was cut to the 600–1700 cm^−1^ wavenumber range and analyzed using the algorithm highlighted in Fig. 1b. The three-step algorithm started with the extraction and subtraction of the baseline fluorescence signal using a polygonal curve (5th degree) approximation. Removing the fluorescence revealed the underlying unknown RR spectrum. The next step of the algorithm fitted pre-recorded RR libraries of reduced and oxidized hemoglobin, along with reduced and oxidized mitochondria onto the unknown spectrum using a regression-based error-minimization approach. This fitting allowed the assignment of weights to individual libraries depending on the concentrations of individual molecules associated with those libraries. These weights called the signal coefficients for individual libraries, were used to calculate the Hemoglobin Index. On the other hand, the weights corresponding to the polygonal approximation for fluorescence were used to calculate the Fluorescence Index.

### Data Collection

Procedure for obtaining Raman spectra from burn wounds - The probe was attached to a 3D-printed adapter, which sits on the skin surface, to maintain a constant distance of 9 mm from the wound and provide a localized dark spot for measurements. Two measurements close to the central location of the wounds were obtained and used for analysis to capture the most representative depth regions from the wounds. Additional measurements were made on four randomly chosen locations on the unwounded healthy skin areas of the back of the animal on the same day for normalization. Normalization accounted for random differences between different devices and systematic error due to systemic differences in hemoglobin levels between animals. This procedure was repeated for each of the eight wounds on three pigs for a total of twelve days of assessment (POD 0, 2, 3, 7, 14, 21, 28, 35, 42, 49, 56, 64) and is summarized in Fig. 1c. It is important to note that on POD7, we only have n = 2 for all four wound categories since the device malfunctioned for two out of the three pigs included in this study for this day. Furthermore, out of these, the 2 wounds in the PT-Superficial category were removed based on depth categorization. Thus, the HI on this day for this category was obtained by averaging the HI for days 3 and 14 for the heatmap analysis.

Procedure for obtaining burn photographs and biopsies for histology – Each burn wound was assessed with photographs for visual, biopsies for histological, and the Raman device for RRS analysis of the wounds. For the visual assessment, photographic images of the back of the pig were obtained using a high-resolution camera under similar lighting conditions on each assessment day. Post-hoc assessment of damage in the early phase (POD0 to 3), and wound healing in the later phase of the study was performed with visual assessment. Representative images from the early phase are shown in Fig. 2a. The biopsy procedure was performed by an experienced surgeon using either a 3- or 4-mm biopsy punch from an area close to the periphery of the wound. The central region was avoided for biopsy to avoid additional damage due to the biopsy procedure and to allow uninhibited healing. All biopsies were fixed in paraformaldehyde for at least 24 hours before transferring to 70% ethanol until the sectioning procedure. The fixed biopsies were embedded in paraffin wax for vertical cross-sectioning to obtain slices that showed features of damage along the entire depth of the skin. One biopsy was obtained from each wound on each day of the assessment for each pig. The vertical cross sections were stained with either Hematoxylin & Eosin or Mason’s Trichrome on a glass slide. All stained slides were digitized using a Hamamatsu Nanozoomer slide scanner under Brightfield settings for depth scoring and other histological assessments. Representative images are shown in Fig. 2b.

### Data Analysis

Classification of wounds into depth categories – Wounds were classified into superficial, PT-Superficial, PT-Deep, and full-thickness burns based on the initial burn-making procedure and visual assessment of the time to heal the wounds. All wounds (n = 4) that were exposed to a 45°C temperature of the block healed by POD 2. These wounds were placed in the superficial category and most closely resembled the classical first-degree burns. All wounds (n = 6) that were exposed to the 96°C temperature of the block looked pale and firm with discoloration and hence were classified as full-thickness burns- closest to the classical third-degree burns. Finally, the wounds created using a block temperature of 63°C were first classified based on the contact duration of the burn-creation device into either superficial partial-thickness (15–20 seconds of contact, n = 8) or deep partial-thickness (30–45 seconds of contact, n = 6). This initial categorization was confirmed with a secondary criterion based on the time to healing of individual wounds (Fig. 2d). Complete healing (i.e., wound closure) before the end of the study (i.e. by POD 56) was chosen as the cut-off for categorization into the PT-Superficial category, while incomplete healing by the end of the study (i.e., POD 64) was the cut-off for categorization into the PT-Deep category. This led to the elimination of 3 wounds that followed the primary criterion but did not fulfill the secondary classification criterion, leading to a final count of n = 6 wounds in the PT-Superficial and n = 5 wounds in the PT-Deep categories (see also **Table S1**).

Scoring of burn depth using histology– Two blinded scorers quantified the burn depth from Trichrome-stained biopsies from post-operative Day 3 obtained before the escharotomy procedure on full-thickness burns. Scoring was performed by measuring the distance from the top of the slice until the end of a damaged vessel or adnexal structure (such as a hair follicle or gland) for five independent measurements per biopsy (see **Fig. S3**). When at least five such structures were not visible, the dark blue coloration of collagen, indicating significant damage to collagen, was used as the secondary indicator of the depth. Additionally, in the case of a missing epidermal layer, an average depth of 100 microns was added for compatibility with measurements from the superficial wounds where the epithelial layer was intact. The average depth for each scorer from the five measurements per biopsy was used for the analysis. Quality control of the obtained scores was performed to improve the assessment accuracy due to the high error rate in histological scoring by even experienced evaluators for burns (39). A cut-off depth of 680 μm was used for the inclusion of scores from observers in the partial-thickness wound categories, based on the commonly accepted top 20% criterion for delimitation of papillary dermis for a skin thickness of roughly 3400 μm observed in other studies (63) and confirmed with our measurements. Based on this criterion, scores above the cut-off value for PT-Superficial wounds and below the cut-off value for PT-Deep wounds were removed from subsequent analyses. Similarly, an inclusion criterion of < 250 μm for the superficial thickness category and > 1750 μm for the full-thickness category was used. This quality control was performed to obtain reliable burn depth scores and did not lead to further discarding of wounds in any category. The final scores are shown in Fig. 2c.

Procedure for obtaining Hemoglobin Index from the Raman spectra – The Hemoglobin Index (or HI) was derived from the Raman spectra using the Signal Coefficients obtained from the regression fitting on the raw spectrum. HI was defined as the sum of the Signal Coefficients of hemoglobin libraries (oxidized and reduced) of each measurement on a wound, normalized by the sum of the same coefficients from healthy skin measurements. The normalization factor was the average of four measurements of healthy skin from the pig that were obtained on the same day of assessment. The following equation for the hemoglobin index can also describe this -

$$\text{H.I}{\text{.}}_{x,y,z}=\frac{{\left({\text{a}}_{HbR}+{\text{b}}_{HbO}\right)}_{x,y,z}}{\sum_{4}{\left({\text{a}}_{HbR}+{\text{b}}_{HbO}\right)}_{healthy,y,z}/4}$$

Where ‘a’ is the Signal Coefficient of Reduced Hemoglobin (HbR), ‘b’ is the Signal Coefficient of Oxidized Hemoglobin (HbO), and the subscripts denote the wound number (x or healthy), pig number (y), and post-operative day (z).

Procedure for obtaining Fluorescence Index from the Raman spectra – The Fluorescence Index (or FI) was derived from the raw spectra by using the coefficients for fluorescence baseline, c (Fig. 1b). FI was defined as the ratio of c for each measurement on a wound, normalized by the average c of four measurements on healthy skin, obtained on the same day of assessment. The following equation for Fluorescence Index can also describe this -

$$F\text{.I}{\text{.}}_{x,y,z}=\frac{{\left({\text{c}}_{Fl}\right)}_{x,y,z}}{\sum_{4}{\left({\text{c}}_{Fl}\right)}_{\text{healthy},y,z}/4}$$

Where ‘a’ is the Signal Coefficient of Reduced Hemoglobin (HbR), ‘b’ is the Signal Coefficient of Oxidized Hemoglobin (HbO), and the subscripts denote the wound number (x or healthy), pig number (y), and post-operative day (z).

Granulation tissue demarcation in histology from POD 64 - A single scorer quantified the granulation tissue depth using histological features in H&E and Trichrome stained slides (representative images shown in Fig. 4d). Granulation tissue was characterized by a lack of criss-cross structure of collagen fibers along with light pink staining in H&E slides, particularly for PT-Deep category burns with mature granulation tissue. The full-thickness burns were characterized by extensive infiltration of fibroblasts and inflammatory cells.

### Statistical Analysis

Replicates and outlier analysis – The two central Raman measurements (HI) from all wounds of a particular category on each day were pooled into a single group for outlier analysis. Outliers were removed using the ROUT method with Q = 2% in GraphPad PRISM software. After removing outliers, the remaining values from the two locations were averaged to generate a unique HI per wound, which was used for further analysis. On POD 3, this led to further removal of n = 1 wounds in the PT-Superficial category and n = 1 wounds in the PT-Deep category from subsequent analysis. Overall, 4 to 6 replicates were used for each wound category for HI and FI assessments.

Statistical methods for comparing HI between different depth categories –This unique HI for each wound on each assessment day was used for analyses described here. The first analysis (Fig. 3a) involved generating a heat map to visually compare the HI intensity across multiple burn depth categories and assessment days. Each cell of the heatmap showed the mean HI (on a color gradient scale of 0–10) for all wounds of a given burn category (y-axis) for a given day (x-axis). This visualization helped identify hotspots and trends in HI. K-means clustering was also performed in RStudio (v**2024.12.1**), as shown on the heatmap, to categorize similarities in HI across time. We then compared differences in the early-mid phase from POD 0 to POD 21 by plotting line graphs (Fig. 3b). Each line of this chart represented the mean HI of all wounds in a given burn category with error bars to represent the standard error of means on each day. Furthermore, a one-way ANOVA was performed to understand the dependence of mean HI on burn depth categories for each assessment day (results highlighted with asterisks for each day). Then, we plotted HIs from PODs 0, 2, and 3 as a bar chart (Fig. 3c–e) to represent mean HI and scattered dots to represent HI for individual wounds. Putting each assessment day on one chart allowed us to directly compare burn categories on each day. We performed a one-way ANOVA with multiple comparisons test (Tukey’s test) to understand significant differences between burn categories on these days. Furthermore, we tested differences in categories on these days using a binary classifier analysis. We plotted receiver operating characteristic curves from the HI values obtained on Day 3 of the procedure before escharotomy (Fig. 3f–h). Each graph represented the ROC curve for classification into the following pairs of burn depth categories- superficial vs partial, partial vs deep-partial, and deep-partial vs full-thickness burns. The area under the curve (AUC) values, along with the standard error and p-value, are provided and shown with each graph. For the final analysis based on HI, we plotted several scatter charts to observe the correlation between HI, histological burn depths, and time to healing. One chart was plotted for each of the following parameters: HI vs depth, HI vs time to healing, and depth vs time to healing. For the time to healing charts, only superficial and superficial partial-thickness wound data was used for correlation due to incomplete healing in deep partial-thickness and full-thickness wounds is unknown. For the HI vs depth chart, superficial wound data is excluded due to the intact epithelial layer of the skin. Each chart also shows the Pearson correlation coefficient and the associated p-values (**Figure S4**).

Statistical methods for comparing FI between different depth categories – A unique FI was obtained similar to HI for each wound and was used for analyses described here. We compared differences in the later phase from POD 28 to POD 64 by plotting line graphs (Fig. 4a). Each line of this chart represented the mean FI of all wounds in a given burn category with error bars to represent the standard error of means on each day. Furthermore, a one-way ANOVA was performed to understand the dependence of mean FI on burn depth categories for each assessment day, and the results were highlighted with asterisks for each day. Then, we plotted FIs from PODs 28 and 64 as a bar chart (Fig. 4b–c) to represent mean FI and scattered dots to represent FI for individual wounds. Putting each assessment day on one chart allowed us to directly compare burn categories on each day. We performed a one-way ANOVA with multiple comparisons test (Tukey’s test) to understand significant differences between burn categories on these days.

All statistical analysis was performed in GraphPad Prism software (v10.4.1) unless mentioned otherwise. The following statistical significance level indicators were used for all graphs- p > 0.05– ns, p < 0.05 - *, p < 0.01 - **, p < 0.001 - ***, p < 0.0001 - ****.

## Supplementary Files

This is a list of supplementary files associated with this preprint. Click to download.

- RamanSpectroscopyforBurnsSupplementary.docx

## Data Availability

All data are available in the main text or the supplementary materials.
